# 39800 Immune Checkpoint Blockade during Periprosthetic Joint Infection

**DOI:** 10.1017/cts.2021.435

**Published:** 2021-03-30

**Authors:** Shay Warren, Greg Charville, Derek Amanatullah

**Affiliations:** Stanford University

## Abstract

ABSTRACT IMPACT: If immune checkpoint blockade increases bacterial clearance with or without antibiotics in vitro, clinical application would be almost immediate and dramatic creating a seismic shift in the current therapeutic paradigm of periprosthetic joint infection. OBJECTIVES/GOALS: Periprosthetic joint infection (PJI) is a major cause of failure after joint replacement. Currently, the treatment of PJI relies on removing biofilm contaminated implants. Some of the bacteria within biofilm undergo a phenotypic shift becoming small colony variants (SCVs). SCVs induce local immunosuppression through PD-1/L1 signaling. METHODS/STUDY POPULATION: We will infect cultured human macrophages and bone marrow aspirate with stable Staphylococcus aureus SVCs and treat with anti-PD-1 or anti-PD-L1 monoclonal antibodies with and without antibiotics (e.g., gentamycin, cefazolin, vancomycin, rifampicin) and assess the residual bacterial viability. We will utilize multiplexed ion beam imaging to quantify PD-1/L1 expression in human tissue from patients with a chronic PJI and compare those to patients undergoing an aseptic revision. Patients with a chronic PJI are likely to have increased expression of PD-1/L1 as their tissue samples are prospectively screened. RESULTS/ANTICIPATED RESULTS: SCVs reduce the phagocytic activity of macrophages and can survive intracellularly. SCVs also induce anti-inflammatory M2-macrophage polarization and recruit a heterogeneous group of immature monocytes and granulocytes called myeloid-derived suppressor cells (MDSC) to the periprosthetic microenvironment. M2-macrophages and MDSCs then produce an immunosuppressive cytokine milieu characterized by increased IL-10 and decreased TNF-α. Clinically isolated SCVs up-regulate the expression of PD-L1 and PD-L2 on the surface of macrophages, representing a mechanism by which SCVs induce host immunosuppression and survive immune clearance. Our preliminary data show PD-L1 expression during septic PJI, but not in aseptic revisions. DISCUSSION/SIGNIFICANCE OF FINDINGS: If immune checkpoint blockade is shown to increase bacterial clearance with or without antibiotics, host immunomodulation would represent a novel class of therapeutic adjuvants to assist surgical debridement and antibiotic administration that could be superimposed on existing treatment algorithms to improve PJI related outcomes.

